# Vibrator-Assisted Start–Stop Exercises Improve Premature Ejaculation Symptoms: A Randomized Controlled Trial

**DOI:** 10.1007/s10508-019-01520-0

**Published:** 2019-11-18

**Authors:** Daniel Ventus, Annika Gunst, Stefan Arver, Cecilia Dhejne, Katarina G. Öberg, Elin Zamore-Söderström, Antti Kärnä, Patrick Jern

**Affiliations:** 1 Department of Psychology, Åbo Akademi University, Fabriksgatan 2, 20500 Turku, Finland; 2 Department of Psychology, University of Turku, Turku, Finland; 3 Anova, Karolinska University Hospital, Stockholm, Sweden; 4 Department of Medicine, Huddinge, Karolinska Institutet, Stockholm, Sweden; 5 Turku, Finland

**Keywords:** Premature ejaculation, Start–stop, Vibrator, Interoceptive awareness, Mindfulness

## Abstract

Premature ejaculation (PE) is associated with decreased quality of life, lower confidence and self-esteem, and higher levels of depression, anxiety, and interpersonal difficulties. Here we investigated the effectiveness of vibrator-assisted start–stop exercises for treatment of PE, and whether the treatment effect could be enhanced by an additional psychobehavioral intervention. Fifty participants with a mean age of 41.7 years were included and randomized into two treatment groups and a waiting list control group. Participants were instructed to perform start–stop exercises while stimulating the penis with a purpose-made vibrator, 3 times a week for 6 weeks. Additionally, participants in one of the treatment groups received additional psychoeducation and performed mindfulness meditation-based body scan exercises three times a week. Data were gathered through online questionnaires before and after treatment, as well as 3 and 6 months after treatment. The interventions reduced PE symptoms with large effect sizes (partial *η*^2^ = .20 across the three groups, *d* [95% CI] = 1.05 [.27, 1.82] and 1.07 [.32, 1.82] for treatment groups compared to waiting list control group). The additional psychobehavioral intervention did not further reduce PE symptoms, but did decrease PE-associated negative symptoms such as levels of sexual distress, anxiety, and depression. No side effects were reported. Vibrator-assisted start–stop exercises can be offered as an adequate treatment option for PE.

**Electronic supplementary material:**

The online version of this article (10.1007/s10508-019-01520-0) contains supplementary material, which is available to authorized users.

## Introduction

Premature ejaculation (PE) is a serious problem associated with a range of adverse psychosocial issues and decreased quality of life (Rosen & Althof, 2008), including anxiety (Dunn, Croft, & Hackett, 1999; Porst et al., 2007; Symonds, Roblin, Hart, & Althof, 2003), depression (Porst et al., 2007), lower confidence and self-esteem (Rowland, Patrick, Rothman, & Gagnon, 2007; Symonds et al., 2003), and personal distress (Giuliano et al., 2008; Patrick et al., 2005; Rowland et al., 2007). Further, PE is also negatively associated with the partner’s sexual satisfaction (Riley & Riley, 2005), partner’s orgasmic frequency (Hartmann, Schedlowski, & Krüger, 2005), and interpersonal difficulties (Giuliano et al., 2008; Patrick et al., 2005; Rowland et al., 2007), including avoiding discussing sexual issues with one’s partner (Rowland et al., 2004), and even refraining from establishing new relationships (Symonds et al., 2003).

According to the fifth edition of the *Diagnostic and Statistical Manual of Mental Disorders* (American Psychiatric Association, 2013), PE can be defined as a persistent pattern of ejaculation occurring during partnered sexual activity within approximately 1 min following vaginal penetration (intravaginal ejaculation latency time, IELT) and before the individual wishes it, provided that the symptom has been present for at least 6 months, is experienced on (almost) all occasions, causes clinically significant distress, and is not better explained by other non-sexual factors. PE can further be divided into lifelong and acquired subtypes (American Psychiatric Association, 2013), with the International Society for Sexual Medicine advocating a 3-min IELT cutoff in the acquired subtype (Althof et al., 2014). While about 20–30% of the population report subjective complaints of premature ejaculation, it is unlikely that more than 4% meet more stringent diagnostic criteria for lifelong PE, including an IELT under 1 min (Althof et al., 2014).

At present, selective serotonin reuptake inhibitors (SSRIs) are considered first-line agents for treatment of PE (McMahon, 2015). However, several studies of the only drug approved for treatment of PE (dapoxetine, an SSRI to be taken on demand) in naturalistic settings have reported high discontinuation rates ranging from 70.6 to 89.6% after 12 months, with common reasons for discontinuation being low efficacy, side effects, cost, and loss of interest in sex (Jern, Johansson, Piha, Westberg, & Santtila, 2014; Mondaini et al., 2013; Park et al., 2017). This indicates that further development of treatment options for PE is warranted.

In addition to pharmacological treatment for PE, psychological–behavioral treatment interventions, such as the start–stop exercise (i.e., repeated manual stimulation of the penis with pauses before reaching ejaculation), are considered attractive treatment options as an alternative to or in conjunction with pharmacological treatment (McMahon, 2015). Start–stop exercises have been proposed to function by deconditioning of learned behavior, altering the ejaculatory reflex, providing an opportunity to explore new sexual techniques, or desensitizing the patient to anxiety that is assumed to cause PE (Grenier & Byers, 1995). One review (Melnik et al., 2011) and a meta-analysis (Cooper, Martyn-St James, Kaltenthaler, Dickinson, & Cantrell, 2015) of existing randomized controlled studies of non-pharmacological treatments have demonstrated that behavioral treatments significantly reduce PE symptoms and importantly do so without incurring side effects. However, the reviews note that research is scarce and methodologically heterogeneous, which in turn limits our ability to make evidence-based recommendations regarding behavioral therapies.

Not much is known regarding the etiology of PE (e.g., Jannini et al., 2015). Penile sensitivity has been suggested as a possible etiological component of PE and has been shown to be associated with PE (Guo et al., 2017; Rowland, Haensel, Blom, & Slob, 1993; Xin et al., 1996, 1997), although other studies contradict these reports (Perretti et al., 2003; Salonia et al., 2009). If penile sensitivity is a cause of PE, penile desensitization could plausibly be a component in the treatment of PE. This notion is robustly supported by the evident ejaculatory latency delaying effects of topical analgesics (Wyllie & Hellstrom, 2011). Desensitization can also be achieved by repeated exposure to stimulation. For example, Herbenick et al. (2009) found that mild genital numbness was reported by women as a result of using a vibrator during masturbation. Further, an experimental laboratory study by Malchaire, Rodriguez Diaz, Piette, Gonçalves Amaral, and de Schaetzen (1998) showed that exposing the hand and arm to vibrations for 30 min led to feelings of numbness and decreased sensory perception.

In line with this hypothesis, Zamar (2012) developed a behavioral treatment for PE where start–stop exercises were performed while stimulating the penis with a vibrating device, resulting in an 11-fold increase in IELT in 61% of the participants after a 6-week training period. These results were partly replicated by Jern (2014), although with less pronounced effects (although it should be noted that the participants in Jern’s study were individuals for whom drug treatment had failed, and thus may have represented a subgroup whose PE symptoms were particularly difficult to treat). Similarly, Rodríguez and López (2016) found that participants reported increased control over ejaculation after completing masturbatory exercises with a device designed for desensitization by adding additional friction and pressure, rather than vibrations.

A second hypothesis regarding the background to PE was suggested by Kaplan (1975), who proposed that PE may be caused by a lack of perception of physical sensations before orgasm, which deprives clients of control over ejaculation. Jern (2014) hypothesized that variation in treatment response might partly be explained by interoceptive awareness (i.e., the sensory awareness that originates from physiological states, processes, actions, and functions; Mehling et al., 2012), so that individuals with low interoceptive awareness may be less receptive to start–stop treatment, in case it may already be too late to take measures to prevent ejaculation once they perceive physiological signals of it approaching. From a cognitive perspective, some PE patients have cognitive distortions such as catastrophizing (Althof, 2016), in light of which physiological signals related to ejaculation can be interpreted as threatening. Patients may therefore actively avoid noticing such physiological signals rather than treating them as useful information. de Carufel and Trudel (2006) incorporated monitoring and modulation of sexual excitement, for example through abdominal breathing and muscle relaxation, in their functional–sexological treatment of PE, and reported tenfold increases in stopwatch-measured ELT. This indicates that psychoeducation regarding listening to and using physiological signals of sexual excitement may be a valuable addition to interventions for treating PE.

Modulation of sexual excitement presupposes awareness of one’s physiological signals, and it would therefore be of interest to improve patients’ general interoceptive body awareness. Borneman, Herbert, Mehling, and Singer (2015) found increases in interoceptive awareness following a 13-week intervention containing weekly group sessions and daily practices of body scan and breath meditation. Silverstein, Brown, Roth, and Britton (2011) found in a randomized trial including 30 female and 14 male undergraduates that female participants’ ability to register bodily responses to sexual stimuli was improved by mindful meditation training, and further noticed improvements in self-reported measures of attention, self-judgment, anxiety, and depression. Thus, if a lack of perception of physical sensations is a cause of PE, mindfulness-based meditation training (i.e., non-judgmental present moment awareness) with focus on improving interoceptive awareness could be a valuable addition to treatment of PE. Mindfulness-based interventions have previously been successfully used for sexual dysfunctions, such as low sexual desire and sexual pain (e.g., Brotto & Basson, 2014; Brotto, Basson, & Luria, 2008; Brotto & Goldmeier, 2015; Gunst et al., 2019; Rosenbaum, 2013); however, studies have thus far used almost exclusively female samples (Goldmeier & Mears, 2010). In a recent pilot study of a mindfulness-based group intervention for men with situational erectile dysfunction, Bossio, Basson, Driscoll, Correia, and Brotto (2018) found that the intervention, which was based on a protocol shown to be effective for female sexual dysfunction, was feasible and the initial results were promising.

The aim of the present study was to investigate the efficacy of vibrator-assisted start–stop exercises for treatment of PE, and whether the treatment effect could be enhanced by an additional psychobehavioral intervention consisting of psychoeducation and training of interoceptive awareness. We hypothesized that both treatment groups would improve more than a waiting list control group on the main outcome measure. Further, the addition of a psychobehavioral intervention was expected to lead to greater improvements in the main outcome measure than the start–stop exercises alone. Treatment effects were hypothesized to be sustained at 3- and 6-month follow-up.

## Method

### Participants

**Fig. 1:**
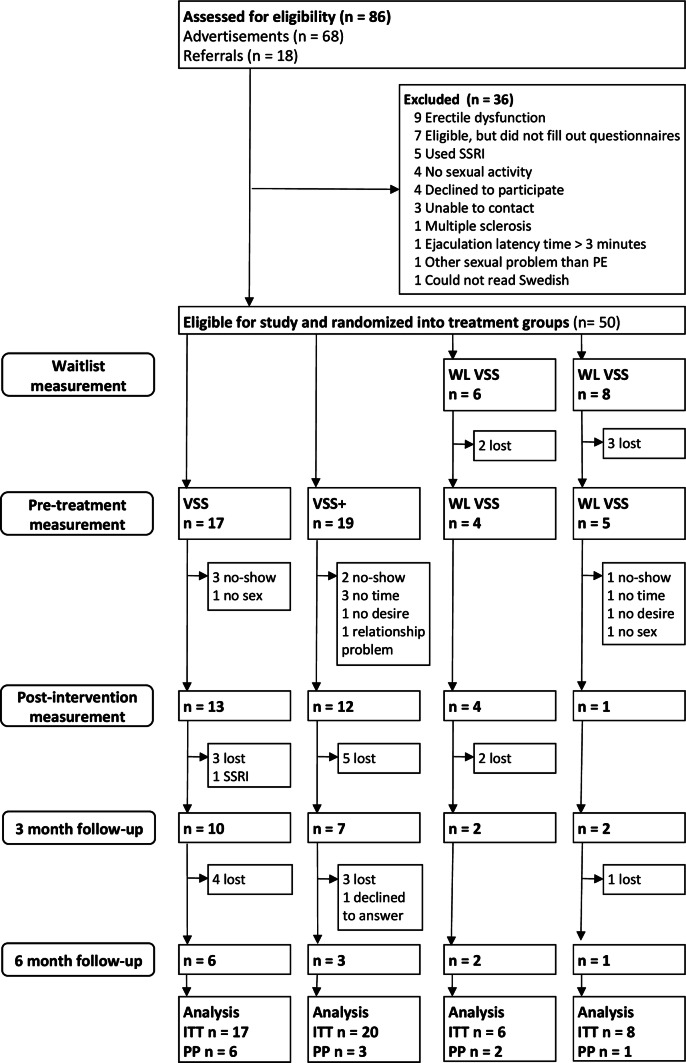
Flowchart of participants in the study. *VSS* vibrator-assisted start–stop group, *VSS+* vibrator-assisted start–stop and psychobehavioral intervention group

Participants were recruited through advertisement in a local newspaper and through referrals to a clinic for sexual medicine at Karolinska University Hospital, Stockholm, Sweden, where the study was conducted. All prospective participants were screened by telephone for the following: Participants had to be at least 18 years old, understand spoken and written Swedish, ejaculate within 3 min after penetration (self-reported), not experience erectile problems that hinder penetrative sex or masturbation, not suffer from multiple sclerosis, and not use medication that may affect ejaculation latency time (ELT), such as SSRIs and opioids. Further, to be eligible for inclusion, participants had to have intercourse with a partner during participation in the study. The ELT cutoff of 3 min was based on the International Society for Sexual Medicine’s definition of acquired PE (Serefoglu et al., 2014). Participants were not screened for other sexual dysfunctions or sexual orientation. See Fig. 1 for a chart of participant flow. The study was carried out from September 2016 to January 2017, with follow-up assessments continuing until July 2017. Regarding sample size, we included all eligible prospective participants who contacted us in time to receive treatment by January 2017.

### Procedure

The study design comprised two treatment groups and a waiting list control condition. The first treatment group performed vibrator-assisted start–stop exercises (VSS), and the second group additionally received a psychobehavioral intervention consisting of psychoeducation and exercises to improve interoceptive awareness (VSS+). Participants were randomized to one of the three groups using a computer-generated random string with a block size of 12. Participants who dropped out, or were excluded after randomization and did not receive allocated intervention, were replaced by subsequently recruited participants to facilitate even group sizes post-treatment. Participants in the control group were further randomized into either treatment group and received treatment following the 6-week waiting period.

The treatment involved two visits to the clinic, 6 weeks apart. The treatment interventions were delivered during the first session, which lasted about 20–30 min for the VSS group and 45 min for the VSS+ group. The interventions were delivered to all participants by the first author, a graduate-level student of clinical psychology. Participants answered online questionnaires used for outcome measures prior to the intervention, immediately after the 6-week intervention, as well as 3 and 6 months post-intervention. Additionally, the control group was assessed at the start of the waiting period (i.e., 6 weeks before the intervention started). The study was approved by the Regional Ethical Review Board in Stockholm, Sweden, in accordance with the Declaration of Helsinki. All participants provided written, informed consent. Participants received treatment, a vibrator, and a bottle of water-based lubricant free of charge. No additional monetary compensation was offered.

#### Interventions

Participants were instructed to complete start–stop exercises using a small handheld vibrator, three times a week for 6 weeks. Participants were instructed to masturbate, holding the lubricated device in contact with the underside of the glans penis, until they felt that ejaculation was imminent. At this point, participants were instructed to move the device away from the penis, and take a break that was long enough for ejaculation to no longer feel imminent, but not so long that they lose erection or desire. This was to be repeated three times, and during the third repetition, participants were instructed to let themselves ejaculate if they so wished. The procedure is based on the manufacturer’s recommendations, and participants were given a translated written version of the instructions. Participants were free to engage in other forms of masturbation or sexual activities as much as they wanted during the 6-week intervention.

In addition to the vibrator-assisted start–stop exercises, participants in the VSS+ group were given further psychoeducation and exercises in interoceptive awareness. The psychoeducation was partly based on the functional–sexological treatment by de Carufel and Trudel (2006), where ejaculation was viewed as a reflex that is triggered once a sufficient level of sexual excitement is reached. Consequently, a goal for treatment was to improve monitoring and control of sexual excitement, mainly by means of improved awareness of bodily reactions during sexual stimulation.

In order to improve interoceptive awareness, participants completed a 15-min guided body scan meditation three times a week, before doing the vibrator-assisted start–stop exercises. Listening to a pre-recorded audio file, participants were instructed to lie or sit down comfortably where they would not be disturbed. The meditation began with an invitation to notice what is going on in the mind and body at the moment, feeling where the body is in contact with the floor or chair, and then focusing on the breath as it enters through the nostrils and goes down into the chest and stomach, observing the breath without controlling it. Staying with the breath, the instructor talked briefly about how the human mind is created to produce an endless stream of thoughts, and how a part of this exercise is to notice when our attention has been carried away by thoughts, and then gently move the attention back to the breath and the present moment. Participants were asked to imagine, if possible, that the body was hollow, and that they could breathe all the way down into their toes. Participants were asked to attend to the sensations in their toes, if any, and to try to shift their attention between different toes and all at once. Subsequently, the attention was shifted to the feet, legs, hips, pelvic floor, genitals, stomach, and upper body. In the end, participants were invited to congratulate themselves for taking the time to get to know their body better, to slowly open their eyes and finish the exercise in their own pace, and to bring this focus and presence into the start–stop exercises. The script for the exercise was inspired by McCown, Reibel, and Micozzi (2010) and recorded by the first author in Swedish.

Participants in the VSS+ group were encouraged to try the following techniques in order to gain better awareness and control over sexual excitement and ejaculation during both start–stop exercises and sexual activities with a partner:

1. Be aware of the level of sexual excitement during all sexual activities;
2. Begin intercourse at a suitable level of excitement, where it is enjoyable, but ejaculation is not imminent;
3. Adjust the level of physical stimulation by taking breaks during intercourse, focusing on other aspects of the sexual exchange than penetration, adjusting tempo of intercourse, and trying different intercourse positions;
4. Pay attention to muscle contractions, especially in the pelvic floor, and try to relax these muscles; and
5. Pay attention to how the breathing changes at different levels of excitement, and try to breathe deeper and slower abdominally.

Participants in both treatment groups were given written instructions and summaries of what was discussed during the first session, as well as a brief diary where they indicated on which days they performed the exercises. See supplementary material for a treatment manual.

### Measures

The Swedish version of the CHecklist for Early Ejaculation Symptoms (CHEES; Dhejne, Jern, & Arver, 2017; Jern, Piha, & Santtila, 2013) is a self-report questionnaire consisting of five questions covering subjectively estimated ejaculation latency time (ELT), feeling of control, feelings of frustration and relationship difficulties as a consequence of rapid ejaculation, and propensity to ejaculate with very little stimulation. CHEES has been validated against stopwatch-measured ELT and other commonly used self-report questionnaires for measuring PE, in both clinical and population-based samples (Jern et al., 2013). The measure was shown to differentiate between diagnosed patients and population-based controls very well (AUC = .98, 95% CI [.97, .98]). Empirically derived cutoffs for CHEES are as follows: 21–25 is strongly indicative of fulfilling diagnostic criteria for PE (correctly identifies 44% of diagnosed PE patients as PE sufferers and incorrectly identifies 1% of controls as PE sufferers), 17–20 is indicative of PE (correctly identifies 90% of diagnosed patients as PE sufferers and incorrectly identifies 5% of controls as PE sufferers), 5–16 points indicate a low probability of PE. In the present study, the reliability of the measure was somewhat low (*α* = .61). This can be expected, as CHEES covers all necessary diagnostic criteria, and these do not necessarily correlate highly (e.g., it is relatively common to report subjective complaints in the absence of very short ELTs).

In addition to the CHEES questions, participants answered the following question regarding possible lifelong problems: “Has it taken you the same amount of time to reach ejaculation all your life, ever since your first intercourse?”

Secondary outcome measures were chosen to measure whether the treatment also had effect on general sexual distress and mental well-being, which is often affected in patients with PE. Furthermore, we wanted to assess the potential etiological role of interoceptive awareness and screen for possible confounding effects of erectile dysfunction.

The Sexual Distress Scale (SDS; Jern et al., 2008) is a self-report questionnaire consisting of seven questions measuring sexual distress using a five-point Likert scale with a total score ranging from 7 to 35. The scale consists of the gender-neutral items of the Female Sexual Distress Scale (Derogatis, Rosen, Leiblum, Burnett, & Heiman, 2002). Example of items included are “How often did you feel: Distressed about your sex life; Sexually inadequate; Embarrassed about sexual problems.” Jern et al. (2008) found the items to load on a single factor with factor loading ranging from .65 to .79 and Cronbach’s *α* = .89. In the present study, the reliability was good (*α* = .86).

The State-Trait Anxiety Inventory (Spielberger, Gorusch, & Lushene, 1970) was used to assess stable (trait) anxiety and situational (state) anxiety. In contrast to the original version, the state anxiety part referred to how the respondents felt the last few times they had sex, instead of how they felt at the moment when answering the questions. Each sum score consists of 20 items, each responded to on a 6-point Likert scale with total scores ranging from 20 to 120. In the present study, the reliability was excellent (*α*_trait_ = .95, *α*_state_ = .93).

The Brief Symptom Inventory-18 (BSI; Derogatis, 2001) is a short screening measure of general psychological distress that was developed from the Symptoms Checklist (SCL-90, Derogatis & Melisaratos, 1983). The subscales of the SCL-90 have previously been shown to possess good discriminant validity in Swedish samples (Fridell, Cesarec, Johansson, & Thorsen, 2002). Participants were instructed to rate how much bother they had experienced during the last 7 days on a 5-point Likert scale, ranging from 0 (not at all) to 4 (very much), with total scores ranging from 0 to 24. We used two of the clinical subscales of the BSI, measuring anxiety and depression, on the basis of the factor structure suggested by Derogatis (2001). The anxiety subscale measures symptoms of nervousness, tension, motor restlessness, apprehension, and panic states. The depression subscale measures anhedonia, loneliness, hopelessness, self-deprecation, and suicidal ideation. In the present study, the reliability was excellent (*α*_anxiety_ = .93, *α*_depression_ = .90).

The Multidimensional Assessment of Interoceptive Awareness (MAIA; Mehling et al., 2012) is a 32-item self-report measure of interoceptive body awareness. MAIA consists of eight subscales: noticing, not-distracting, not-worrying, attention regulation, emotional awareness, self-regulation, body listening, and trusting (*αs* = .59–.91). MAIA has been shown to distinguish between groups with more and less experience of mind–body therapies and is correlated with other questionnaires measuring mindful attention and body awareness (Mehling et al., 2012). Studies of divergent validity indicate that the construct assessed by MAIA is not related to anxiety or anxiety-associated hypervigilance (Mehling, 2016). The factor structure has been confirmed in multiple independent samples (Mehling, 2016).

The five-item version of the International Index of Erectile Function (Rosen, Cappelleri, Smith, Lipsky, & Peña, 1999) was used to measure presence and severity of erectile dysfunction. The measure was included primarily as a screening instrument, as erectile problems were grounds for exclusion from the study. The items cover the ability to achieve and maintain an erection hard enough for penetration, as well as satisfaction with intercourse. In the present study, the reliability was good (*α* = .82).

### Statistical Analyses

Both per-protocol and intention-to-treat results are reported. In the present study, per-protocol analyses include only participants who did not drop out of the study. In intention-to-treat analyses, the last observation was carried forward for cases with missing data. The intention-to-treat analyses are conservative in pre-post analyses, in the sense that participants who do not provide data at post-treatment are assumed to have had no change, which may diminish effect sizes. On the other hand, intention-to-treat analyses can overestimate long-term effects, since any potential treatment effect among participants who drop out is assumed to be sustained.

Normality was assessed using visual inspection of diagnostic plots. Differences between groups after randomization were analyzed using analysis of variance and chi-square. Dropout analyses were conducted in the same fashion, comparing individuals who dropped out to individuals who completed treatment. Associations between study variables at baseline were analyzed using bivariate correlations. Differences between pre- and post-treatment were tested using analysis of covariance, with post-treatment scores being the dependent variable, treatment being the between-group factor (waiting list, VSS, VSS+), and pre-treatment measurement used as a covariate in order to reduce error variance (Dimitrov & Rumrill, 2003). Partial *η*^2^ effect sizes were calculated for the ANCOVA and were interpreted as small (*η*^2^ = .01), medium (*η*^2^ = .06), or large (*η*^2^ = .14; Cohen, 1988). Cohen’s *d* and confidence intervals were calculated for pairwise comparisons between groups, based on the adjusted post-means estimated in the ANCOVA, and interpreted as small (*d* = .20), medium (*d* = .50), or large (*d* = .80; Cohen, 1988). For inferential statistics, *p* < .05 was considered statistically significant. Within-group differences were calculated using dependent *t* tests. Differences between the two treatment groups at follow-up were tested using independent *t* tests. A change score was calculated for PE by subtracting pre-scores from post-scores. Cronbach’s *α* was calculated for all measures at the first time point to examine reliability. All statistical analyses were conducted with SPSS for Windows and Mac, version 24.0.

## Results

Of 86 assessed individuals, 50 were eligible for inclusion in the study and were randomized into treatment or control groups (see Fig. 1). Thirty participants provided data post-treatment, 21 at 3-month and 12 at 6-month follow-up. No statistically significant differences were found between the three groups at the first measurement, except for one MAIA subscale, indicating a successful randomization (see Table 1 for demographic variables and supplementary tables S1 and S2 for significance testing). There was a statistically significant correlation between PE and sexual distress at the first measurement (*r* = .49, *p* < .001, see supplementary table S3). PE was not statistically significantly correlated with any other measure.

**Table 1:** Demographic variables

Variable	Group		
	Waiting list	VSS	VSS+
Age (in years) *M* (SD)	43.43 (9.12)	41.59 (9.33)	40.42 (12.10)
Height (cm) *M* (SD)	180.57 (5.67)	182.71 (7.95)	181.32 (6.29)
Weight (kg) *M* (SD)	85.86 (14.38)	80.71 (12.54)	81.68 (12.59)
No. children *M* (SD)	2.00 (1.18)	1.76 (1.03)	1.58 (1.02)
Relationship duration (years) *M* (SD)	13.67 (11.46)	13.12 (8.12)	10.79 (14.31)
How many times have you had sex with a partner during the last month? *M* (SD)	3.57 (2.41)	2.29 (1.61)	3.63 (3.67)
How many times would you have wanted to have sex with a partner during the last month? *M* (SD)	8.57 (5.12)	6.88 (5.16)	8.32 (4.96)
Education (*n*, %)			
Primary	1 (7.7)	1 (5.9)	4 (23.5)
Secondary	4 (30.8)	3 (17.6)	3 (17.6)
University or equivalent	6 (46.2)	13 (765)	8 (47.1)
Other	2 (15.4)	0 (0)	2 (11.8)
Sexual orientation (*n*, %)			
Strictly heterosexual	13 (100)	15 (88.2)	17 (89.5)
More heterosexual than homosexual	0 (0)	2 (11.8)	2 (10.5)
Equally heterosexual and homosexual	0 (0)	0 (0)	0 (0)
More homosexual than heterosexual	0 (0)	0 (0)	0 (0)
Strictly homosexual	0 (0)	0 (0)	0 (0)
Marital status (*n*, %)			
No partner	1 (7.7)	0 (0)	2 (10.5)
Married or cohabiting	10 (16.9)	16 (94.1)	12 (63.2)
In a relationship but not cohabiting	2 (15.4)	1 (5.9)	5 (26.3)
Lifelong PE			
Yes	3 (21.4)	4 (23.5)	3 (15.8)
No	11 (78.6)	13 (76.5)	16 (84.2)

No statistically significant differences between groups (see supplementary material). Lifelong PE = subjective experience of always having had an ejaculation latency time ≤ 1 min

### Pre-Post Analyses with Control Group

Means and SDs of study variables are presented in Table 2. Within-group *t* tests for dependent groups showed that the control group had no statistically significant changes on any of the variables during the waiting period (Table 3). The VSS group had a significant improvement in PE symptoms (i.e., decline in scores on the composite PE symptom variable) between pre- and post-treatment measures, but no reductions in variables measuring other symptoms. The VSS+ group, however, reported statistically significant reductions in symptoms of PE, sexual distress, and all three measures of anxiety at post-treatment.

**Table 2:** Means and SDs at each measurement point for each group

	WL (*N *= 14)		VSS (*N *= 17)				VSS+ (*N *= 19)			
	Pre	Post	Pre	Post	3 mo	6 mo	Pre	Post	3 mo	6 mo
Intention-to-treat with the last observation carried forward										
PE	20.35 (3.05)	20.86 (2.91)	20.41 (2.87)	18.35 (3.89)	17.82 (5.27)	17.41 (5.97)	21.00 (2.40)	18.79 (3.08)	18.26 (3.62)	17.95 (3.96)
SDS	21.21 (5.07)	21.07 (4.45)	19.06 (4.99)	19.88 (6.12)	18.29 (7.93)	17.65 (6.78)	22.47 (5.39)	19.05 (5.84)	19.74 (6.31)	19.16 (6.92)
STAI-T	52.36 (17.92)	53.07 (17.30)	48.82 (13.59)	48.00 (14.05)	47.29 (14.67)	45.24 (14.47)	55.26 (17.87)	48.74 (12.29)	49.74 (14.26)	49.16 (14.37)
STAI-S	51.36 (17.42)	53.86 (17.23)	51.35 (13.79)	51.35 (16.92)	47.76 (15.03)	46.94 (18.75)	59.00 (12.22)	52.37 (13.46)	51.21 (14.43)	49.53 (15.84)
BSI-A	8.79 (2.33)	9.00 (2.39)	8.24 (2.80)	9.06 (2.97)	8.76 (3.58)	9.00 (3.43)	11.37 (6.69)	8.79 (3.41)	9.58 (4.89)	9.53 (5.06)
BSI-D	10.21 (3.77)	10.64 (3.95)	9.35 (3.86)	9.94 (3.47)	9.53 (3.56)	9.47 (2.76)	12.05 (6.56)	9.16 (2.77)	9.26 (3.18)	9.16 (3.27)
ED	24.57 (3.84)	24.64 (3.46)	25.56 (2.34)	25.71 (2.78)	26.06 (2.19)	25.65 (5.05)	24.82 (3.91)	25.29 (3.08)	26.24 (1.99)	26.12 (2.00)

	WL		VSS				VSS+			
	Pre (*N *= 14)	Post (*N *= 9)	Pre (*N *= 17)	Post (*N *= 13)	3 mo (*N* = 10)	6 mo (*N* = 6)	Pre (*N* = 19)	Post (*N* = 12)	3 mo (*N* = 7)	6 mo (*N* = 3)
Per-protocol										
PE	20.35 (3.05)	21.33 (3.24)	20.41 (2.87)	18.54 (4.10)	16.40 (5.72)	22.17 (2.04)	21.00 (2.40)	17.67 (3.11)	16.14 (3.29)	13.00 (1.73)
SDS	21.21 (5.07)	21.78 (4.68)	19.06 (4.99)	19.77 (6.87)	16.00 (8.06)	22.17 (5.71)	22.47 (5.39)	19.08 (6.75)	18.71 (5.38)	12.00 (3.61)
STAI-T	52.36 (17.92)	48.00 (14.39)	48.82 (13.59)	43.15 (7.63)	42.50 (11.30)	43.17 (12.04)	55.26 (17.87)	52.75 (13.31)	50.43 (16.57)	42.67 (6.51)
STAI-S	51.36 (17.42)	53.89 (17.76)	51.35 (13.79)	49.77 (17.21)	43.90 (14.05)	50.17 (25.33)	59.00 (12.22)	54.17 (16.41)	47.14 (15.52)	30.33 (2.08)
BSI-A	8.79 (2.33)	9.33 (2.65)	8.24 (2.80)	8.38 (2.43)	6.90 (.99)	8.83 (1.94)	11.37 (6.69)	9.83 (3.90)	10.86 (6.12)	8.67 (4.62)
BSI-D	10.21 (3.77)	10.44 (3.47)	9.35 (3.86)	8.92 (2.14)	8.70 (3.53)	10.00 (1.79)	12.05 (6.56)	9.67 (3.06)	9.14 (3.76)	8.33 (2.31)
ED	24.57 (3.84)	24.89 (2.62)	25.56 (2.34)	25.77 (2.62)	26.40 (1.51)	23.67 (7.94)	24.82 (3.91)	24.58 (3.40)	26.86 (1.78)	27.33 (2.08)

*ED* erectile dysfunction, *PE* premature ejaculation, *SDS* sexual distress, *STAI*-*T* trait anxiety subscale of State-Trait Anxiety Inventory, *STAI*-*S* state anxiety (during sex) subscale of State-Trait Anxiety Inventory, *BSI*-*A* anxiety subscale of Brief Symptom Inventory, *BSI*-*D* depression subscale of Brief Symptom Inventory, *WL* waiting list, *VSS* vibrator-assisted start–stop, *VSS+* vibrator-assisted start–stop and psychobehavioral intervention, *pre* before intervention, *post* after intervention, *3 mo* 3-month follow-up, *6 mo* 6-month follow-up

**Table 3:** Testing of differences before and after treatment within and between groups

	Within group						Between group					
	WL (*N* = 14)		VSS (*N* = 17)		VSS+ (*N* = 19)		ANCOVA			Pairwise comparisons of post-scores *d* [95% CI]		
	*t*	*p*	*t*	*p*	*t*	*p*	*F*	*p*	Partial *η*^2^	VSS versus WL	VSS+ versus WL	VSS+ versus VSS
Intention-to-treat with last observation carried forward												
Premature ejaculation	.92	.373	**3.22**	**.005**	**3.78**	**.001**	**5.65**	**.006**	**.20**	**1.05 [.27, 1.82]**	**1.07 [.32, 1.82]**	.02 [− .65, .70]
Sexual distress	.10	.919	1.10	.288	**2.48**	**.023**	2.21	.122	.09	− .03 [− .76, .69]	.60 [− .12, 1.32]	.63 [− .06, 1.32]
STAI Trait	.39	.701	.62	.542	**2.77**	**.013**	2.21	.122	.09	− .03 [− .76, .69]	.60 [− .12, 1.32]	.63 [− .06, 1.32]
STAI State during sex	1.39	.187	.00	1.00	**2.18**	**.043**	1.43	.250	.06	.21 [− .52, .94]	.58 [− .14, 1.30]	.38 [− .30, 1.06]
BSI Anxiety	.90	.385	1.41	.177	**2.17**	**.044**	2.14	.130	.08	− .12 [− .85, .61]	.54 [− .17, 1.26]	.66 [− .03, 1.36]
BSI Depression	.73	.481	.89	.385	2.05	.055	2.65	.082	.10	.13 [− .59, .86]	**.74 [.02, 1.47]**	.61 [− .08, 1.30]

	Within group						Between group					
	WL (*N* = 9)		VSS (*N* = 13)		VSS+ (*N* = 12)		ANCOVA			Pairwise comparisons of post-scores *d* [95% CI]		
	*t*	*p*	*t*	*p*	*t*	*p*	F	p	Partial *η*^2^	VSS versus WL	VSS+ versus WL	VSS+ versus VSS
Per-protocol												
Premature ejaculation	.92	.385	**3.57**	**.004**	**5.07**	**.000**	**7.34**	**<.001**	**.33**	**1.31 [.34, 2.28]**	**1.63 [.59, 2.67]**	.31 [− .51, 1.14]
Sexual distress	.10	.922	1.10	.292	**2.72**	**.020**	2.07	.140	.12	− .01 [− .89, .87]	.76 [− .18, 1.69]	.75 [− .10, 1.60]
STAI Trait	.39	.710	.62	.547	**3.14**	**.009**	1.21	.310	.07	.39 [− .50, 1.28]	.70 [− .23, 1.63]	.32 [− .50, 1.14]
STAI State during sex	1.42	.192	.00	1.00	**2.33**	**.040**	1.05	.360	.07	.27 [− .61, 1.15]	.65 [− .27, 1.58]	.39 [− .43, 1.22]
BSI Anxiety	.89	.397	1.43	.179	**2.31**	**.041**	.42	.660	.03	.13 [− .75, 1.01]	.41 [− .50, 1.32]	.27 [− .55, 1.10]
BSI Depression	.72	.493	.89	.391	2.17	.053	.77	.470	.05	.44 [− .45, 1.33]	.49 [− .42, 1.41]	.06 [− .75, .88]

Cohen’s *d* effect sizes are computed from estimated post-measurement scores adjusted for pre-measurement in the ANCOVA. Statistically significant tests are bolded

*STAI* State-Trait Anxiety Inventory, *BSI* Brief Symptom Inventory, *WL* waiting list, *VSS* vibrator-assisted start–stop, *VSS+* vibrator-assisted start–stop and psychobehavioral intervention

**Fig. 2:**
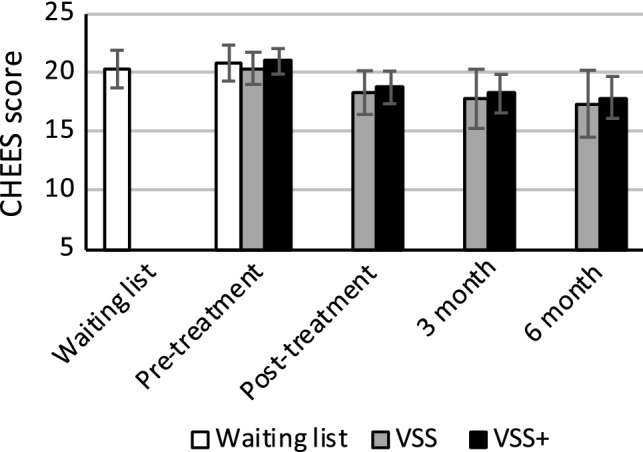
Premature ejaculation scores from intention-to-treat analyses. *N*_VSS_ = 23, *N*_VSS+_ = 27. Empirically derived cutoff scores: 21–25: strongly indicative of fulfilling diagnostic criteria for PE, 17–20: indicative of PE, 5–16: low probability of PE. *CHEES* CHecklist for Early Ejaculation Symptoms, *VSS* vibrator-assisted start–stop group, *VSS+* vibrator-assisted start–stop and psychobehavioral intervention group. Error bars represent 95% confidence intervals

**Fig. 3:**
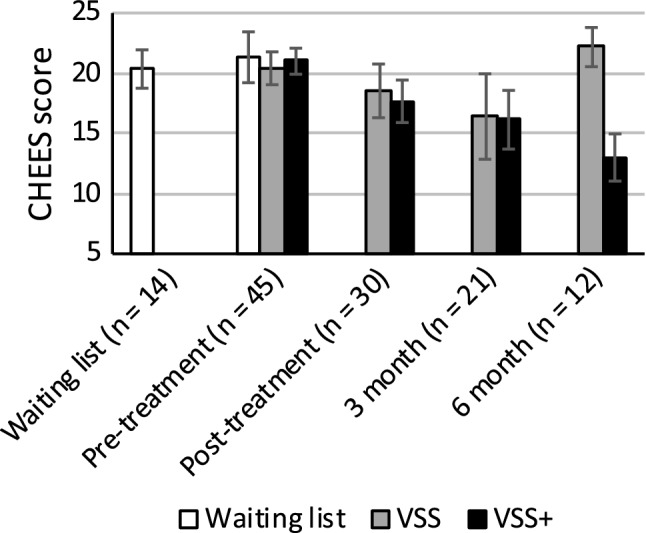
Premature ejaculation scores from per-protocol analyses. Empirically derived cutoff scores: 21–25: strongly indicative of fulfilling diagnostic criteria for PE, 17–20: indicative of PE, 5–16: low probability of PE. *CHEES* CHecklist for Early Ejaculation Symptoms, *VSS* vibrator-assisted start–stop group, *VSS+* vibrator-assisted start–stop and psychobehavioral intervention group. Error bars represent 95% confidence intervals

ANCOVA analyses of post-scores for PE, including pre-scores as a covariate, showed that there were statistically significant differences between the three groups (waiting list, VSS, VSS+), with large effect sizes in both per-protocol analyses (partial *η*^2^ = .33) and intention-to-treat analyses (partial *η*^2^ = .20; see Table 3 and Figs. 2 and 3). ANCOVA analyses revealed no other statistically significant between-group differences. The same pattern of statistical significance was found in within- and between-group intention-to-treat and per-protocol analyses, with effect sizes being slightly larger in per-protocol analyses.

Pairwise comparisons of the groups showed that treatment effects for PE in both treatment groups had large effect sizes compared to the control group (*d*_VSS_ = 1.05, *d*_VSS+_ = 1.07). Regarding PE, the treatment groups did not differ from one another statistically significantly, nor in terms of effect size, at the post-intervention measurement. When comparing secondary outcome measures between treatment groups, while not statistically significant, the VSS+ had larger treatment effects than the VSS group on sexual distress, STAI trait anxiety, and both BSI measures, with medium effect sizes (*d *= .61–.66). The effect was less pronounced for STAI anxiety during sex (*d* = .38).

Treatment effect in terms of participants’ categorization on the main outcome measure is descriptively presented in Table 4. Looking at the most severe category, a larger change can be observed in the VSS+ group than in the VSS group. A majority of the participants in the VSS group did not change category, while three quarters of the VSS+ group showed improvement in one or two categories.

**Table 4:** Categorization of participants according to CHEES scores before and after treatment across groups (*n*)

CHEES		Group			
		VSS		VSS+	
Score	Categorization	Pre	Post	Pre	Post
21–25	Strongly indicative of fulfilling diagnostic criteria for PE	11	9	8	1
17–20	Indicative of PE	5	1	5	6
5–16	Low probability of PE	1	7	1	7

Data presented for participants who answered both pre- and post-treatment questionnaires

*CHEES* CHecklist for Early Ejaculation Symptoms, *PE* premature ejaculation, *Pre* pre-treatment, *Post* post-treatment, *VSS* vibrator-assisted start–stop, *VSS+* vibrator-assisted start–stop and psychobehavioral intervention

We also performed a post hoc ANCOVA analysis of the treatment effect using a sum score of only the CHEES items measuring sexual function (ejaculation latency time, feeling of control, ejaculation with very little stimulation) at post-treatment as the dependent variable, including pre-scores as a covariate. The intention-to-treat analysis revealed a statistically significant difference between the three groups (*F* = 6.39, *p* = .004, partial *η*^2^ = .22). Pairwise comparisons between groups revealed large effect sizes for both the VSS group (*d* = 1.26, 95% CI [.47, 2.06]) and the VSS+ group (*d* = .96, 95% CI [.22, 1.71]) when compared to the control group. The VSS group had a slightly larger effect on sexual functioning than the VSS+ group (*d* = .30, 95% CI [− .98, .37]), but the difference was not statistically significant.

In a further exploratory step, we analyzed if having lifelong PE affected the treatment effect across the entire sample. An ANCOVA analysis of post-scores for PE, including pre-scores as a covariate, using lifelong PE as a grouping variable, showed that there was no statistically significant difference between participants who did and did not report always having had an ELT less than 1 min (*F* = 1.59, *p* = .214, partial *η*^2^ = .03).

### Dropout

Between the pre- and post-measurements, 11 participants dropped out from the treatment groups. Five participants dropped out from the control group during the waiting period. There was no statistically significant difference in dropout rate between groups (*p* = .65). When comparing mean differences on the primary outcome measure between participants who did and did not drop out on all variables at the first measurement, no statistically significant differences were found (all *p*_s_ > .09).

In order to analyze whether dropout at 3-month follow-up could be explained by previous treatment effect, we compared the PE change score (post-scores minus pre-scores) between participants who did and did not provide data at 3-month follow-up. There was no statistically significant difference between the groups (*M*_complete_ = − 2.65, *SD*_complete_ = 2.43, *M*_dropout_ = − 2.80, SD_dropout_ = 2.94, *t*(28) = .149, *p* = .883).

### Treatment Adherence

Among participants who returned the exercise diary (*n *= 26), the mean number of vibrator-assisted start–stop exercises was 2.37 per week in the VSS group and 1.88 in the VSS+ group (*t*(23) = 1.50, *p* = .147), suggesting that some participants did not complete the training protocol as instructed. There was no statistically significant correlation between the number of uses of the vibrator and change in PE score between pre- and post-measurement (*r* = .22, *p* = .279).

### Follow-Up at 3 and 6 months

Participants from the waiting list were included in one of the two treatment groups in the follow-up analyses. No statistically significant differences were found between the two treatment groups at either follow-up time (Table 5). The numbers of responses at 3 and 6 months were 12 and 8 for the VSS group, and 9 and 4 for the VSS+ group, respectively. Intention-to-treat analyses revealed that improvements in PE symptoms were sustained after 3 and 6 months in the VSS group, when compared to pre-treatment. Likewise, levels of PE were significantly lower at 3 and 6 months in the VSS+ group compared to pre-treatment. Further, the VSS+ group reported sustained improvements in sexual distress, STAI trait and state at 3 and 6 months, as well as lower depression scores at 6 months compared to pre-treatment. However, as the last observation was carried forward for participants with missing data in the intention-to-treat analyses, effects might be overestimated and should be interpreted with caution. Per-protocol analyses revealed greater disparities between the groups at 6 months, with the VSS group returning to baseline levels of PE and the VSS+ group further improving. These analyses were, however, based on a small number of observations, and thus, these results should be interpreted with caution.

**Table 5:** Testing of within-group differences between follow-up and baseline and between-group differences at follow-up

	VSS (*N* = 23)						VSS+ (*N* = 27)						Between-group differences					
	Pre versus 3 mo			Pre versus 6 mo			Pre versus 3 mo			Pre versus 6 mo			3 mo (*N* = 48–50)			6 mo (*N* = 48–50)		
	*t*	*df*	*p*	*t*	*df*	*p*	*t*	*df*	*p*	*t*	*df*	*p*	*t*	*df*	*p*	*t*	*df*	*p*
Intention-to-treat with last observation carried forward																		
Premature ejaculation	**2.249**	**22**	**.035**	**2.236**	**22**	**.036**	**4.043**	**26**	**<.001**	**3.874**	**26**	**.001**	.209	48	.835	.499	48	.620
Sexual distress	.358	22	.724	.957	22	.349	**2.375**	**26**	**.025**	**2.816**	**26**	**.009**	.931	48	.356	1.097	48	.278
STAI Trait	.543	22	.593	1.424	22	.168	**3.089**	**26**	**.005**	**3.070**	**26**	**.005**	1.203	48	.235	1.463	48	.150
STAI State during sex	.702	22	.490	1.232	22	.231	**2.521**	**26**	**.018**	**2.700**	**26**	**.012**	.689	48	.494	.579	48	.565
BSI Anxiety	.891	22	.382	1.854	22	.077	1.333	26	.194	1.394	26	.175	1.011	48	.317	.697	48	.489
BSI Depression	.109	22	.914	.075	22	.941	2.020	26	.054	**2.155**	**26**	**.041**	.931	48	.356	.953	48	.345

	VSS						VSS+						Between-group differences					
	Pre versus 3 mo (*N* = 12)			Pre versus 6 mo (*N* = 8)			Pre versus 3 mo (*N* = 9)			Pre versus 6 mo (*N* = 4)			3 mo (*N* = 21)			6 mo (*N* = 12)		
	*t*	*df*	*p*	*t*	*df*	*p*	*t*	*df*	*p*	*t*	*df*	*p*	*t*	*df*	*p*	*t*	*df*	*p*
Per-protocol																		
Premature ejaculation	**2.489**	**11**	**.030**	.369	7	.723	**6.934**	**8**	**<.001**	**3.538**	**3**	**.038**	1.096	19	.287	2.186	10	.054
Sexual distress	1.105	11	.293	.685	7	.515	**3.223**	**8**	**.012**	**6.481**	**3**	**.007**	.444	18.32	.662	1.981	10	.076
STAI Trait	.333	11	.745	.707	7	.502	**3.613**	**8**	**.007**	**3.189**	**3**	**.050**	.800	19	.433	.054	10	.958
STAI State during sex	**2.484**	**11**	**.030**	.475	7	.649	**3.538**	**8**	**.008**	**7.533**	**3**	**.005**	.103	19	.919	1.626	8.77	.139
BSI Anxiety	.432	11	.674	**3.035**	**7**	**.019**	.910	8	.390	2.109	3	.126	1.872	8.37	.096	.309	10	.764
BSI Depression	.060	11	.953	.092	7	.929	1.681	8	.131	1.470	3	.238	.362	17.45	.722	.872	10	.403

Statistically significant tests are presented in bold typeface

*VSS* vibrator-assisted start–stop, *VSS+* vibrator-assisted start–stop and psychobehavioral intervention, *STAI* State-Trait Anxiety Inventory, *BSI* Brief Symptom Inventory, *pre* before intervention, *3 mo* 3-month follow-up, *6 mo* 6-month follow-up

### Interoceptive Awareness

At baseline, the VSS+ group reported statistically significantly lower scores on the Trusting subscale of MAIA than the VSS group (*M*_VSS+_ = 8.37, SD_VSS+_ = 3.93; *M*_VSS_ = 11.30, SD_VSS_ = 2.74; *t*(48) = 3.01, *p* = .004, see supplementary table S2). At no other time point did the groups differ statistically significantly on any subscale. This means that that the body scan intervention did not have the anticipated effect of improving IA in the VSS+ group.

## Discussion

In accordance with our hypothesis, we found that a 6-week intervention consisting of vibrator-assisted start–stop exercises significantly reduced self-reported PE symptoms. Compared to a waiting list control group, the two treatment groups displayed improvements of large effect sizes on the primary outcome measure at post-treatment. Large treatment effect sizes were also found when looking at only the items of the measure that pertain to sexual functioning (ELT, feeling of control, frequency of ejaculation with little stimulation). Looking at change in term of categorization on the main outcome measure, 58% of all participants improved by one or two categories between pre- and post-treatment. These results are in line with previous studies demonstrating the potential effectiveness of the intervention. Jern (2014) included 11 participants in a pilot study of the vibrator-assisted start–stop intervention, reporting a medium standardized mean difference on CHEES between pre- and post-treatment (*d* = .71). This is comparable to the corresponding within-group effect size for the VSS group in the present study (*d* = .54). Zamar (2012) also reported an 11-fold improvement in latency period in 61% of a sample completing the same device-assisted 6-week start–stop intervention. Taken together, these studies provide replicated results, indicating that the vibrator-assisted start–stop technique significantly improves PE symptoms.

In the present study, there were no statistically significant differences in terms of PE symptoms at any point between the VSS and the VSS+ groups, indicating that the treatment effect of vibrator-assisted start–stop exercises on PE symptoms is, contrary to our hypothesis, not further enhanced by the psychobehavioral intervention. However, with regard to change in categorization on the primary outcome measure between pre- and post-treatment results favor the VSS+ group, as 11 of 14 (79%) of the VVS+ sample improved by one or two categories, while the corresponding numbers for the VSS group were 7 out of 17 (41%).

Also contrary to our expectations, the VSS+ group did not report higher scores on MAIA scales than the VSS group at any point post-intervention. Thus, we are unable to directly test whether improved interoceptive awareness would reduce PE symptoms. In a previous study, Borneman et al. (2015) found increases in five out of eight MAIA scales (*d*s ranging from .2 to .7) following a 13-week intervention containing weekly group sessions and daily practices of body scan and breath meditation. It is conceivable that the exercises performed by the participants in the present study were not sufficient to bring about improved interoceptive awareness.

Unlike the VSS group, the VSS+ group improved statistically significantly on measures of sexual distress, trait anxiety, and state anxiety during sex, compared to baseline. Comparisons between the two groups on secondary outcome measures, while not statistically significant, revealed medium effect sizes indicating larger improvements in the VSS+ group. As such, the added elements of psychoeducation and exercises related to interoceptive awareness made the treatment more holistic, addressing not only PE, but also reducing problems related to PE and improving mental well-being. The lack of statistically significant differences may be due to low statistical power.

At baseline, PE was not statistically significantly correlated with anxiety or depression. Further, the change in PE was similar in both treatment groups, despite statistically significant decreases in anxiety only within the VSS+ group. While previous cross-sectional studies have found associations between anxiety and PE (e.g., Dunn et al., 1999), a recent longitudinal study found no causal associations over time (Ventus, Gunst, Kärnä, & Jern, 2017), which is in line with the present results.

No side effects were reported. Eighteen participants dropped out between pre- and post-treatment. Ten of these gave reasons, a lack of time on the part of the participant being the most common (see Fig. 1). It is possible that the extent of the treatment protocol was unnecessarily large, as a treatment effect was found even though the mean number of exercises performed per week was lower than instructed. With fewer exercises per week ordinated, the treatment effect might have been the same, while the dropout rate possibly would have been lower. Two participants reported having no desire, sexual or otherwise, to complete the exercises. There were no statistically significant differences at baseline between participants who did and did not provide data at post-treatment, and no difference in pre- to post-treatment effect for PE between those who did and did not supply data at 3-month follow-up. While it is possible that participants who dropped out without giving a reason for it might have done so because of poor treatment effect or side effects, the absence of systematic differences between individuals who completed the treatment and individuals who did not is a strength of the present study, as the results are less likely skewed by dropout.

Nevertheless, the small sample size reduces our power to find actual effects and increases the risk of spurious results (Button et al., 2013), which is a major limitation of the present study. Another limitation of the present study was that all participants answered 32 items regarding interoceptive awareness at each measurement point. This might in itself be a sort of interoceptive awareness intervention by letting all participants know that interoceptive awareness is of interest. As such, the VSS group might become more like the VSS+ group than intended, leading to a decreased internal validity. Further, the two treatment groups got somewhat different amounts of attention from the clinician at the first meeting (about 20 min vs. 45 min), which could affect treatment response (Freedland, Mohr, Davidson, & Schwartz, 2011); the improvements in secondary outcome measures in the VSS+ group could be explained by either the additional psychobehavioral intervention or the additional attention by the clinician. A major limitation of the study design is that we are not able to discern between effects of the different components in the additional psychobehavioral intervention. Treatment adherence with regard to the additional psychobehavioral component was not assessed, and as such, it is possible that the VSS and VSS+ groups did not differ due to participants in the VSS+ group not performing the recommended body scan exercises. Another limitation was that treatment was delivered by the first author only, and treatment fidelity was not assessed; having multiple clinicians providing treatment, and assessing whether they adhere to the treatment manual, would have reduced the risk of potential biases (such as the clinician’s preconceived hypothesis of which treatment will be more effective) and would have shown generalizability of treatment effect across clinicians. The ecological validity of the present findings is limited due to exclusion criteria (e.g., no erectile dysfunction, no use of SSRI) making the sample unrepresentative of the entire population of men with PE.

Future studies could include measures of penile sensitivity, in order to elucidate whether the hypothesized mechanism of action is correct. To test hypotheses regarding the effect of improved interoceptive awareness on PE, future studies could employ a more intensive face-to-face-guided interoceptive awareness training program. Further, future studies could examine the quality of the romantic relationship, and whether working on it in the intervention improves treatment effect. The sampling frame could also be expanded to include participants who suffer from some degree of concurrent erectile problems, to elucidate whether the treatment effect generalizes to this group of patients.

The present study provided further evidence that behavioral methods can lead to significant reductions in PE, with a majority of the participants in the present study improving in terms of their categorization on a validated scale of PE. While the present first-line treatment of PE is associated with a range of side effects, contributing to making treatment with SSRIs unacceptable to most patients, (Jern et al., 2014; Mondaini et al., 2013; Park et al., 2017), no side effects have been reported by the vibrator-assisted start–stop exercises in the present or previous studies, besides complaints concerning difficulty to find time to complete all recommended exercises (Jern, 2014; Zamar, 2012). As for the practical administration of treatment, taking a pill is simple and quick, whereas start–stop exercises necessitate a considerable investment of time and effort. On the other hand, as seen in the present study, the effect of the behavioral treatment might be sustained, whereas the effect of pharmacological aids is not. Further, dapoxetine must be taken 1–3 h before anticipated intercourse, which may also be perceived as inconvenient in that it does not allow for completely spontaneous initiations of partnered sexual activities. Taken together, there are good reasons to offer behavioral treatments as an adequate alternative to pharmacotherapy for patients seeking treatment for PE.

## Electronic supplementary material

Below is the link to the electronic supplementary material.

Supplementary material 1 (DOCX 56 kb)

Supplementary material 2 (DOCX 58 kb)

## Data Availability

The raw data associated with the present study are available at: osf.io/bw6uh.
