# Supplementary material for: Vibrator-Assisted Start–Stop Exercises Improve Premature Ejaculation Symptoms: A Randomized Controlled Trial
Source: Arch Sex Behav. 2019 Nov 18;49(5):1559–73. doi: 10.1007/s10508-019-01520-0 (PMC7300103; doi:10.1007/s10508-019-01520-0)
Supplement: Supplementary file 1 — Supplementary material 1 (DOCX 56 kb) [file 10508_2019_1520_MOESM1_ESM.docx]

Start-stop exercises with vibrator and interoceptive awareness training for treating premature ejaculation: a randomized waiting list controlled study

Treatment manual

Daniel Ventus, Annika Gunst, Stefan Arver, Cecilia Dhejne, Elin Zamore-Söderström, Katarina Görts Öberg, Patrick Jern

During the first meeting, practical details of participation in the study are discussed. The treatment commence by presenting statistics on the mean intercourse duration (about 4-5 minutes), to break the ice and debunk wrongful beliefs. Participants are then interviewed in order to outline their specific situation and build report. After this, one group receive only instructions to do start-stop exercises with vibrator, while the other group additionally receive psychoeducation and training in interoceptive awareness.

Interview and discussion

| Topic | Example questions |
| --- | --- |
| Outlining of the dysfunction  *Are there any experiences of control?* | How long does it take between when you first enter your partner with your penis and when you ejaculate?  Has it been this way all your life?  Has the time varied with different partners?  How long does it take during masturbation? |
| Previous treatment attempts  *Instill hope* | What have you tried to do previously to solve the problem?  What has and has not worked?  Pros and cons with different treatment options  Why did you seek help right now? |
| How is the romantic relationship affected by PE  *For whose sake do you want to change?* | Does your partner think that PE is a problem?  Have you talked about it?  Do you talk about sex with your partner? |
| Intercourse frequency, sexual repertoire  *Avoidance behavior?*  *How many opportunities for learning?* | How often do you have sex?  Can you describe a typical sexual episode?  Do you engage in other sexual activities than penetrative intercourse?  Does your partner usually orgasm? |
| Thoughts and feelings during sex  *Presence during sex* | Can you describe the last time you had sex?  What thoughts went through your head before/during/after sex?  How did you feel before/during/after sex? |

Start-stop exercise with vibrator

Participants are instructed to complete start-stop exercises using a small handheld vibrator, three times a week for six weeks. A Prolong vibrator (Auris medtech Europe ltd., Eastbourne, UK), which has been developed specifically for treatment of PE, along with a tube of water-based lubricant is provided to each participant. Participants are instructed to masturbate, holding the lubricated device in contact with the underside of the glans penis, until they feel that ejaculation is imminent. At this point, participants are instructed to move the device away from the penis, and take a break that is long enough for ejaculation to no longer feel imminent, but not so long that they lose erection or desire. This is to be repeated three times, and during the third repetition, participants were can let themselves ejaculate. The instructions follow the manufacturer’s recommendations.

We discuss the possibility that they sometimes might not be able to stop themselves in time. In this case, seeing as they have passed the point of no return, they are instructed to let themselves enjoy the ejaculation.

The rationale for the exercises is explained as twofold:

1. The starting and stopping is an old technique that can help some men learn to control their ejaculation
2. The vibration desensitizes the penis, meaning that more stimulation (time) is necessary during intercourse in order to reach ejaculation.

Psychoeducation

The psychoeducation is partly based on the functional-sexological treatment by de Carufel and Trudel^1^, which is based on the following premises:

- Ejaculation is a reflex that cannot be controlled
- The reflex is triggered once sexual excitement reaches a sufficient level of intensity
- The level of sexual excitement can be controlled voluntarily (to some extent).

Consequently, a goal for treatment is to improve monitoring and control of sexual excitement, mainly by means of improved awareness of bodily reactions during sexual stimulation.


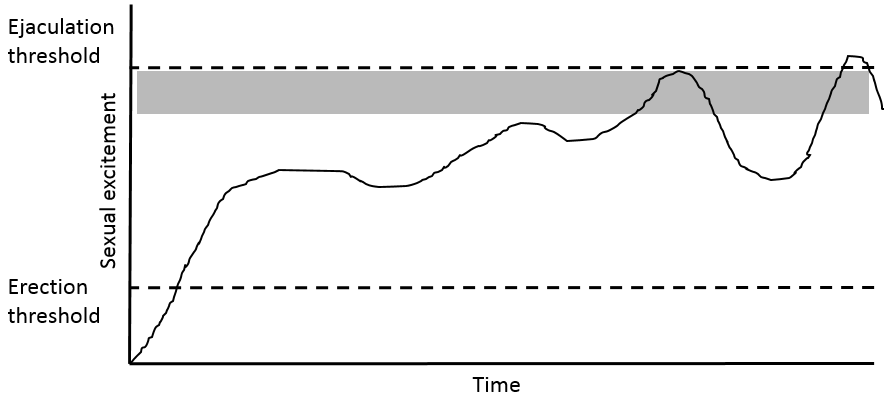


The figure illustrates how a person’s sexual excitement might change during a sexual encounter. The gray area before the ejaculation threshold is important, because the body can give signals that one is getting closer to ejaculation. In order to facilitate recognition of bodily signals that indicate that ejaculation is near, the following examples can be given: increased muscle tension, faster breathing, holding of breath, increased tempo of intercourse, increased feelings of stimulation, increased pleasure, stiffening of the penis, testicles being pulled up towards the body, increased heart rate, and blushing. The stronger these signals, the closer to ejaculation. Which signals that are present varies from person to person, every one must identify what is present in their own body. There may be other signs or signals not noted above.

Learning to identify one’s position on the excitement scale is a central goal of treatment. The start-stop exercises provide an opportunity to listen to the body, and learn to regulate the level of excitement. Further, if the vibrations have a desensitizing effect, this would mean that the threshold for ejaculation is not reached as easily, giving more space in the appropriate middle level of the excitement scale. In a sense, the threshold for ejaculation is “pushed upwards” in the figure, giving more space.

Participants are instructed to turn their attention to their body during all forms of sexual stimulation, rather than the often-used strategy of distracting oneself in order to decrease sexual excitement. The rationale for this advice is that it is not possible to control the body if you do not know what is happening in the body. While distracting oneself from sexual stimuli might reduce a subjective experience of sexual excitement, it does not necessarily decrease the bodily sexual arousal. In other words, there might be a discrepancy between body and mind in the level of sexual excitement, and the goal of the present treatment is to become more attuned to the bodily aspect.

Regulating sexual excitement

The following techniques can be used in order to gain better awareness of and control over sexual excitement and ejaculation. The techniques can be practiced during both start-stop exercises (masturbation) and sexual activities with a partner. Every sexual experience can be seen as an opportunity to increase knowledge of one’s sexual responses.

1. Pay attention to the level of sexual excitement during all sexual activity. Being aware of what is happening in the body facilitates being able to control what happens in the body.
2. Begin intercourse at a suitable level of excitement, where it is enjoyable but ejaculation is not imminent. If the level of excitement is too high from the start, there is no time or opportunity to control ejaculation.
3. Adjust the level of physical stimulation. This can be achieved by taking breaks during intercourse, adjusting tempo of intercourse, and trying different intercourse positions. This is also related to sexual repertoire, one can focus on other aspects of the sexual exchange than penetration, such as caressing the body, hugging, or giving oral sex.
4. Pay attention to muscle contractions, especially in the pelvic floor, and try to relax these muscles. Contracting these muscles may sometimes be a spontaneous reaction when one tries to “hold back” the ejaculation, but this may have an opposite effect. Since pelvic floor muscle contraction is part of the ejaculation mechanism, relaxation of these muscles might counteract ejaculation. Studies have shown that exercises of pelvic floor muscle awareness and control can have an ejaculation-delaying effect.^2,3^
5. Pay attention to how the breathing changes at different levels of excitement. Some men notice that they hold their breath when they get excited. Others say they start breathing shallowly and quickly. Patients can be advised to try first notice how their breath changes with excitement, and then to breathe deeper and slower abdominally. Breathing in this way may have a calming effect on the body.

Training interoceptive awareness

To train body awareness, we first discuss being present in the moment. The following may serve as a brief example:

*Thinking is a fantastic human skill. It allows us to learn from what has happened before and try to predict what might happen in the future. We have thousands of thoughts every day. And while it is great that we can think, it can sometimes hinder us from being present in the moment. For example, when you are about to have sex, you might think back at previous experiences where sex has not worked out as you had hoped. This thought makes you anxious “What if it happens again? This time will also be a failure”. Here already we see the past and the anticipated future at play, making it difficult to appreciate the present moment. With these thoughts about performance and risk failure, it is hard to appreciate the intimacy, perhaps love, how the other person’s skin feels against your skin. Turning the attention to what is happening in this moment also turns the attention to what is happening in the body at this moment. And as we have discussed previously, this may facilitate knowing and affecting what is happening in the body.*

Participants are given a link to a 15-minute long audio file of a body-scan meditation recorded by the first author, based on McCown et al^4^. The instructions guide the listener to attend to the breath, systematically turning their attention to different parts of the body, and reminding the listener to move the attention gently back to the task whenever they notice their mind getting distracted. And this is the exercise: to move the attention back again and again. Noticing that their attention has diverted is not a failure, but a success. Participants are instructed to meditate lying down in a comfortable position where they will not be disturbed, and listen to the audio file. This is to be completed three times a week for six weeks. Ideally, this meditation exercise would be completed just before doing start-stop exercises with the vibrator, as this would facilitate body awareness during the start-stop exercise.

References

1. de Carufel F, Trudel G. Effects of a New Functional-Sexological Treatment for Premature Ejaculation. *J Sex Marital Ther*. 2006;32(731694649):97-114. doi:10.1080/00926230500442292.

2. Pastore AL, Palleschi G, Fuschi A, et al. Pelvic floor muscle rehabilitation for patients with lifelong premature ejaculation: a novel therapeutic approach. *Ther Adv Urol*. 2014;6:83-88. doi:10.1177/1756287214523329.

3. Pastore AL, Palleschi G, Leto A, et al. A prospective randomized study to compare pelvic floor rehabilitation and dapoxetine for treatment of lifelong premature ejaculation. *Int J Androl*. 2012;35(1):528-533. doi:10.1111/j.1365-2605.2011.01243.x.

4. McCown D, Reibel D, Micozzi M. *Teaching Mindfulness: A Practical Guide for Clinicians and Educators*. New York, NY: Springer; 2010.
