# Supplementary material for: Vibrator-Assisted Start–Stop Exercises Improve Premature Ejaculation Symptoms: A Randomized Controlled Trial
Source: Arch Sex Behav. 2019 Nov 18;49(5):1559–73. doi: 10.1007/s10508-019-01520-0 (PMC7300103; doi:10.1007/s10508-019-01520-0)
Supplement: Supplementary file 2 — Supplementary material 2 (DOCX 58 kb) [file 10508_2019_1520_MOESM2_ESM.docx]

Table S1

Descriptive statistics, demographics and comparisons between groups at the first measurement

|  |  |  |  |  |  |  | Group differences | | |
| --- | --- | --- | --- | --- | --- | --- | --- | --- | --- |
| Variable | Waiting list  (*n* = 12-13) |  | VSS  (*n* = 16-17) |  | VSS+BA  (*n* = 18-20) |  | *F* | *χ2* (*df*) | *p* |
| Premature ejaculation *M* (*SD*) | 20.35 (3.05) |  | 20.41 (2.87) |  | 21.00 (2.40) |  | 0.29 |  | .75 |
| STAI Trait *M* (*SD*) | 21.21 (5.07) |  | 19.06 (4.99) |  | 22.47 (5.39) |  | 0.68 |  | .51 |
| STAI State during sex *M* (*SD*) | 52.36 (17.92) |  | 48.82 (13.59) |  | 55.26 (17.87) |  | 1.67 |  | .20 |
| Erectile function *M* (*SD*) | 51.36 (17.42) |  | 51.35 (13.79) |  | 59.00 (12.22) |  | 0.35 |  | .71 |
| Sexual distress *M* (*SD*) | 8.79 (2.33) |  | 8.24 (2.80) |  | 11.37 (6.69) |  | 1.98 |  | .15 |
| BSI Anxiety *M* (*SD*) | 10.21 (3.77) |  | 9.35 (3.86) |  | 12.05 (6.56) |  | 2.35 |  | .11 |
| BSI Depresison *M* (*SD*) | 24.57 (3.84) |  | 25.56 (2.34) |  | 24.82 (3.91) |  | 1.35 |  | .27 |
| Age *M* (*SD*) | 43.43 (9.12) |  | 41.59 (9.33) |  | 40.42 (12.10) |  | 0.34 |  | .72 |
| Height (cm) *M* (*SD*) | 180.57 (5.67) |  | 182.71 (7.95) |  | 181.32 (6.29) |  | 0.41 |  | .67 |
| Weight (kg) *M* (*SD*) | 85.86 (14.38) |  | 80.71 (12.54) |  | 81.68 (12.59) |  | 0.66 |  | .52 |
| No. Children *M* (*SD*) | 2.00 (1.18) |  | 1.76 (1.03) |  | 1.58 (1.02) |  | 0.63 |  | .54 |
| Relationship duration (years) *M* (*SD*) | 13.67 (11.46) |  | 13.12 (8.12) |  | 10.79 (14.31) |  | 0.29 |  | .75 |
| How many times have you had sex with a partner during the last month? *M* (*SD*) | 3.57 (2.41) |  | 2.29 (1.61) |  | 3.63 (3.67) |  | 1.26 |  | .29 |
| How many times would you have wanted to have sex with a partner during the last month? *M* (*SD*) | 8.57 (5.12) |  | 6.88 (5.16) |  | 8.32 (4.96) |  | 0.53 |  | .59 |
| ADHD *M* (*SD*) | 2.00 (1.53) |  | 1.18 (1.29) |  | 1.39 (1.61) |  | 1.19 |  | .31 |
| Education (*n*, %) |  |  |  |  |  |  |  | 7.26 (6) | .30 |
| Primary | 1 (7.7) |  | 1 (5.9) |  | 4 (23.5) |  |  |  |  |
| Secondary | 4 (30.8) |  | 3 (17.6) |  | 3 (17.6) |  |  |  |  |
| University or equivalent | 6 (46.2) |  | 13 (765) |  | 8 (47.1) |  |  |  |  |
| Other | 2 (15.4) |  | 0 (0) |  | 2 (11.8) |  |  |  |  |
| Sexual orientation (n, %) |  |  |  |  |  |  |  | 1.59 (2) | .45 |
| Strictly heterosexual | 13 (100) |  | 15 (88,2) |  | 17 (89.5) |  |  |  |  |
| More heterosexual than homosexual | 0 (0) |  | 2 (11,8) |  | 2 (10.5) |  |  |  |  |
| Marital status (n, %) |  |  |  |  |  |  |  | 5.11 (4) | .28 |
| No partner | 1 (7.7) |  | 0 (0) |  | 2 (10.5) |  |  |  |  |
| Married or cohabiting | 10 (16.9) |  | 16 (94.1) |  | 12 (63.2) |  |  |  |  |
| In a relationship but not cohabiting | 2 (15.4) |  | 1 (5.9) |  | 5 (26.3) |  |  |  |  |
| Lifelong PE |  |  |  |  |  |  |  | 0.36 (2) | .84 |
| Yes | 3 (21.4) |  | 4 (23.5) |  | 3 (15.8) |  |  |  |  |
| No | 11 (78.6) |  | 13 (76.5) |  | 16 (84.2) |  |  |  |  |

*Note.* VSS = vibrator-assisted start-stop, VSS+BA = vibrator-assisted start-stop and body awareness, STAI = State-Trait Anxiety Inventory, BSI = Brief Symptom Inventory, ADHD = attention deficit hyperactivity disorder, Lifelong PE = subjective experience of always having had an ejaculation latency time ≤ 1 minute.

Table S2. Descriptive statistics and within and between group t-tests for all MAIA subscales

| Means and standard deviations for MAIA subscales at each time point, per treatment group | | | | | | | | | |
| --- | --- | --- | --- | --- | --- | --- | --- | --- | --- |
|  |  | **Pre** | | **Post** | | **3 mo** | | **6 mo** | |
| Group |  | Mean | Std. Deviation | Mean | Std. Deviation | Mean | Std. Deviation | Mean | Std. Deviation |
| VSS (N = 23) | Noticing | 10,22 | 4,51 | 11,30 | 4,23 | 10,39 | 5,46 | 10,87 | 4,79 |
|  | Not-distracting | 9,09 | 3,26 | 8,70 | 3,62 | 8,91 | 3,60 | 8,43 | 3,19 |
|  | Not-worrying | 9,04 | 2,27 | 8,83 | 2,93 | 8,78 | 2,50 | 9,00 | 2,07 |
|  | Attention regulation | 14,91 | 6,91 | 15,13 | 6,24 | 14,52 | 7,54 | 16,91 | 6,91 |
|  | Emotional awareness | 13,17 | 4,56 | 14,43 | 5,22 | 13,83 | 5,45 | 14,78 | 5,62 |
|  | Self regulation | 8,65 | 3,52 | 9,00 | 3,37 | 8,57 | 4,39 | 9,17 | 3,81 |
|  | Body listening | 4,48 | 3,55 | 4,48 | 3,03 | 4,13 | 3,33 | 5,35 | 4,15 |
|  | Trusting | 11,30 | 2,74 | 11,04 | 3,23 | 11,48 | 3,80 | 11,04 | 3,90 |
| VSS+BA (N = 27) | Noticing | 9,41 | 4,83 | 10,81 | 4,24 | 10,74 | 4,38 | 10,52 | 4,45 |
|  | Not-distracting | 8,26 | 2,75 | 8,37 | 2,44 | 8,22 | 2,61 | 8,63 | 2,65 |
|  | Not-worrying | 8,00 | 2,96 | 8,74 | 2,70 | 8,70 | 2,81 | 8,85 | 2,61 |
|  | Attention regulation | 16,33 | 6,71 | 17,30 | 6,74 | 17,44 | 6,90 | 17,37 | 6,93 |
|  | Emotional awareness | 13,26 | 5,31 | 13,96 | 4,89 | 14,52 | 5,18 | 14,30 | 5,07 |
|  | Self regulation | 8,93 | 3,43 | 9,67 | 3,61 | 9,74 | 3,74 | 9,70 | 3,74 |
|  | Body listening | 5,11 | 3,14 | 5,37 | 3,08 | 5,70 | 3,21 | 5,81 | 3,37 |
|  | Trusting | 8,37 | 3,93 | 9,67 | 3,51 | 9,48 | 3,65 | 9,44 | 3,60 |

*continued*

|  | Between group differences, independent t-tests | | | | | | | | | | | | | | |
| --- | --- | --- | --- | --- | --- | --- | --- | --- | --- | --- | --- | --- | --- | --- | --- |
|  | Pre | | |  | Post | | |  | 3 mo | | |  | 6 mo | | |
|  | t | df | p |  | t | df | p |  | t | df | p |  | t | df | p |
| Noticing | .609 | 48 | .545 |  | .407 | 48 | .686 |  | -.251 | 48 | .803 |  | .268 | 48 | .789 |
| Not-distracting | .973 | 48 | .335 |  | .366 | 37.498 | .717 |  | .784 | 48 | .437 |  | -.236 | 48 | .814 |
| Not-worrying | 1.380 | 48 | .174 |  | .107 | 48 | .915 |  | .104 | 48 | .918 |  | .220 | 48 | .827 |
| Attention regulation | -.736 | 48 | .465 |  | -1.172 | 48 | .247 |  | -1.431 | 48 | .159 |  | -.233 | 48 | .817 |
| Emotional awareness | -.060 | 48 | .952 |  | .329 | 48 | .743 |  | -.460 | 48 | .647 |  | .322 | 48 | .749 |
| Self regulation | -.278 | 48 | .782 |  | -.671 | 48 | .505 |  | -1.023 | 48 | .311 |  | -.495 | 48 | .623 |
| Body listening | -.668 | 48 | .507 |  | -1.029 | 48 | .309 |  | -1.697 | 48 | .096 |  | -.439 | 48 | .663 |
| Trusting | **3.008** | **48** | **.004** |  | 1.435 | 48 | .158 |  | 1.891 | 48 | .065 |  | 1.507 | 48 | .138 |

| Paired Samples Test PRE-POST | |  |  |  |  |  |  |  |  |
| --- | --- | --- | --- | --- | --- | --- | --- | --- | --- |
|  |  | Paired Differences | |  | 95% Confidence Interval of the Difference | | |  |  |
| Group |  | Mean | Std. Deviation | Std. Error Mean | Lower | Upper | t | df | Sig. (2-tailed) |
| VSS | MAIA_noticingPRE - MAIA_noticingPOST | -1.08696 | 3.20388 | .66805 | -2.47242 | .29850 | -1.627 | 22 | .118 |
|  | MAIA_notDistractPRE - MAIA_notDistractPOST | .39130 | 2.12644 | .44339 | -.52824 | 1.31085 | .883 | 22 | .387 |
|  | MAIA_notWorryPRE - MAIA_notWorryPOST | .21739 | 2.35404 | .49085 | -.80057 | 1.23535 | .443 | 22 | .662 |
|  | MAIA_attRegPRE - MAIA_attRegPOST | -.21739 | 2.57535 | .53700 | -1.33105 | .89627 | -.405 | 22 | .690 |
|  | MAIA_emoAwarPRE - MAIA_emoAwarPOST | -1.26087 | 3.40077 | .70911 | -2.73147 | .20973 | -1.778 | 22 | .089 |
|  | MAIA_selfRegPRE - MAIA_selfRegPOST | -.34783 | 1.61270 | .33627 | -1.04521 | .34956 | -1.034 | 22 | .312 |
|  | MAIA_bodyListPRE - MAIA_bodyListPOST | .00000 | 1.70561 | .35564 | -.73756 | .73756 | .000 | 22 | 1.000 |
|  | MAIA_trustPRE - MAIA_trustPOST | .26087 | 1.98213 | .41330 | -.59627 | 1.11801 | .631 | 22 | .534 |
| VSS+BA | MAIA_noticingPRE - MAIA_noticingPOST | -1.40741 | 3.71338 | .71464 | -2.87637 | .06156 | -1.969 | 26 | .060 |
|  | MAIA_notDistractPRE - MAIA_notDistractPOST | -.11111 | 1.15470 | .22222 | -.56790 | .34567 | -.500 | 26 | .621 |
|  | MAIA_notWorryPRE - MAIA_notWorryPOST | -.74074 | 1.65466 | .31844 | -1.39530 | -.08618 | -2.326 | 26 | .028 |
|  | MAIA_attRegPRE - MAIA_attRegPOST | -.96296 | 5.04961 | .97180 | -2.96052 | 1.03460 | -.991 | 26 | .331 |
|  | MAIA_emoAwarPRE - MAIA_emoAwarPOST | -.70370 | 3.43975 | .66198 | -2.06443 | .65702 | -1.063 | 26 | .298 |
|  | MAIA_selfRegPRE - MAIA_selfRegPOST | -.74074 | 3.18159 | .61230 | -1.99934 | .51785 | -1.210 | 26 | .237 |
|  | MAIA_bodyListPRE - MAIA_bodyListPOST | -.25926 | 2.15893 | .41549 | -1.11330 | .59478 | -.624 | 26 | .538 |
|  | **MAIA_trustPRE - MAIA_trustPOST** | **-1.29630** | **3.01043** | **.57936** | **-2.48718** | **-.10541** | **-2.237** | **26** | **.034** |
|  |  |  |  |  |  |  |  |  |  |
|  |  |  |  |  |  |  |  |  |  |
| Paired Samples Test PRE-3MO | |  |  |  |  |  |  |  |  |
|  |  | Paired Differences | |  | 95% Confidence Interval of the Difference | | |  |  |
| Group |  | Mean | Std. Deviation | Std. Error Mean | Lower | Upper | t | df | Sig. (2-tailed) |
| VSS | MAIA_noticingPRE - MAIA_noticingPOST | -1.08696 | 3.20388 | .66805 | -2.47242 | .29850 | -1.627 | 22 | .118 |
|  | MAIA_notDistractPRE - MAIA_notDistractPOST | .39130 | 2.12644 | .44339 | -.52824 | 1.31085 | .883 | 22 | .387 |
|  | MAIA_notWorryPRE - MAIA_notWorryPOST | .21739 | 2.35404 | .49085 | -.80057 | 1.23535 | .443 | 22 | .662 |
|  | MAIA_attRegPRE - MAIA_attRegPOST | -.21739 | 2.57535 | .53700 | -1.33105 | .89627 | -.405 | 22 | .690 |
|  | MAIA_emoAwarPRE - MAIA_emoAwarPOST | -1.26087 | 3.40077 | .70911 | -2.73147 | .20973 | -1.778 | 22 | .089 |
|  | MAIA_selfRegPRE - MAIA_selfRegPOST | -.34783 | 1.61270 | .33627 | -1.04521 | .34956 | -1.034 | 22 | .312 |
|  | MAIA_bodyListPRE - MAIA_bodyListPOST | .00000 | 1.70561 | .35564 | -.73756 | .73756 | .000 | 22 | 1.000 |
|  | MAIA_trustPRE - MAIA_trustPOST | .26087 | 1.98213 | .41330 | -.59627 | 1.11801 | .631 | 22 | .534 |
| VSS+BA | MAIA_noticingPRE - MAIA_noticingPOST | -1.40741 | 3.71338 | .71464 | -2.87637 | .06156 | -1.969 | 26 | .060 |
|  | MAIA_notDistractPRE - MAIA_notDistractPOST | -.11111 | 1.15470 | .22222 | -.56790 | .34567 | -.500 | 26 | .621 |
|  | **MAIA_notWorryPRE - MAIA_notWorryPOST** | **-.74074** | **1.65466** | **.31844** | **-1.39530** | **-.08618** | **-2.326** | **26** | **.028** |
|  | MAIA_attRegPRE - MAIA_attRegPOST | -.96296 | 5.04961 | .97180 | -2.96052 | 1.03460 | -.991 | 26 | .331 |
|  | MAIA_emoAwarPRE - MAIA_emoAwarPOST | -.70370 | 3.43975 | .66198 | -2.06443 | .65702 | -1.063 | 26 | .298 |
|  | MAIA_selfRegPRE - MAIA_selfRegPOST | -.74074 | 3.18159 | .61230 | -1.99934 | .51785 | -1.210 | 26 | .237 |
|  | MAIA_bodyListPRE - MAIA_bodyListPOST | -.25926 | 2.15893 | .41549 | -1.11330 | .59478 | -.624 | 26 | .538 |
|  | **MAIA_trustPRE - MAIA_trustPOST** | **-1.29630** | **3.01043** | **.57936** | **-2.48718** | **-.10541** | **-2.237** | **26** | **.034** |
|  |  |  |  |  |  |  |  |  |  |
|  |  |  |  |  |  |  |  |  |  |
| Paired Samples Test PRE_6MO | |  |  |  |  |  |  |  |  |
|  |  | Paired Differences | |  | 95% Confidence Interval of the Difference | | |  |  |
| Group |  | Mean | Std. Deviation | Std. Error Mean | Lower | Upper | t | df | Sig. (2-tailed) |
| VSS | MAIA_noticingPRE - MAIA_noticing_6MO | -.65217 | 4.11879 | .85883 | -2.43327 | 1.12892 | -.759 | 22 | .456 |
|  | MAIA_notDistractPRE - MAIA_notDistract_6MO | .65217 | 2.75695 | .57486 | -.54002 | 1.84437 | 0,13472222 | 22 | .269 |
|  | MAIA_notWorryPRE - MAIA_notWorry_6MO | .04348 | 2.14218 | .44668 | -.88287 | .96983 | .097 | 22 | .923 |
|  | MAIA_attRegPRE - MAIA_attReg_6MO | -2.00000 | 5.40202 | 1.12640 | -4.33601 | .33601 | -1.776 | 22 | .090 |
|  | **MAIA_emoAwarPRE - MAIA_emoAwar_6MO** | **-1.60870** | **3.68953** | **.76932** | **-3.20417** | **-.01322** | **-2.091** | **22** | **.048** |
|  | MAIA_selfRegPRE - MAIA_selfReg_6MO | -.52174 | 3.46239 | .72196 | -2.01899 | .97551 | -.723 | 22 | .477 |
|  | MAIA_bodyListPRE - MAIA_bodyList_6MO | -.86957 | 2.94347 | .61376 | -2.14242 | .40329 | -1.417 | 22 | .171 |
|  | MAIA_trustPRE - MAIA_trust_6MO | .26087 | 3.00329 | .62623 | -1.03785 | 1.55959 | .417 | 22 | .681 |
| VSS+BA | MAIA_noticingPRE - MAIA_noticing_6MO | -1.11111 | 2.96561 | .57073 | -2.28427 | .06205 | -1.947 | 26 | .062 |
|  | MAIA_notDistractPRE - MAIA_notDistract_6MO | -.37037 | 2.67680 | .51515 | -1.42928 | .68854 | -.719 | 26 | .479 |
|  | **MAIA_notWorryPRE - MAIA_notWorry_6MO** | **-.85185** | **1.74761** | **.33633** | **-1.54318** | **-.16052** | **-2.533** | **26** | **.018** |
|  | MAIA_attRegPRE - MAIA_attReg_6MO | -1.03704 | 4.86338 | .93596 | -2.96092 | .88685 | -1.108 | 26 | .278 |
|  | MAIA_emoAwarPRE - MAIA_emoAwar_6MO | -1.03704 | 2.95455 | .56860 | -2.20582 | .13174 | -1.824 | 26 | .080 |
|  | MAIA_selfRegPRE - MAIA_selfReg_6MO | -.77778 | 3.00427 | .57817 | -1.96623 | .41067 | -1.345 | 26 | .190 |
|  | MAIA_bodyListPRE - MAIA_bodyList_6MO | -.70370 | 2.43081 | .46781 | -1.66530 | .25789 | -1.504 | 26 | .145 |
|  | **MAIA_trustPRE - MAIA_trust_6MO** | **-1.07407** | **2.47955** | **.47719** | **-2.05495** | **-.09320** | **-2.251** | **26** |  |

The VSS+BA group displayed significant increases in the *trusting* subscale at post-treatment, three-month, and six-month follow-up when compared to baseline, and significant increases in the *not-worrying* subscale at both follow-ups when compared to baseline. The VSS group reported significantly higher on the *emotional awareness* subscale at six-month follow-up compared to baseline. The analyses at six months were, however, based on a small number of observations and should therefore be interpreted with caution.

Table S3

Correlations between study variables at first measurement (*N* = 50)

| Variable | 1. | 2. | 3. | 4. | 5. | 6. | 7. |
| --- | --- | --- | --- | --- | --- | --- | --- |
| 1. Premature ejaculation | - |  |  |  |  |  |  |
| 2. Sexual distress | .486*** | - |  |  |  |  |  |
| 3. STAI trait | .051 | .372** | - |  |  |  |  |
| 4. STAI state during sex | .174 | .422** | .640*** | - |  |  |  |
| 5. BSI anxiety | .062 | .442** | .729*** | .553*** | - |  |  |
| 6. BSI depression | .079 | .462** | .772*** | .520*** | .849*** | - |  |
| 7. Erectile function^a^ | -.196 | -.463** | -0.263 | -.429** | -.375** | -.376** | - |

Note . STAI = State-Trait Anxiety Inventory, BSI = Brief Symptom Inventory

* = *p* < .05

** = *p* < .01

*** = *p* < .001

^a^ *n* = 47, higher scores indicate better erectile function

There was a statistically significant correlation between PE and sexual distress at the first measurement. PE was not statistically significantly correlated with any other measure. All measures of anxiety, depression and distress were positively correlated, with correlation coefficients ranging from .372 to .849. Erectile function was negatively correlated to all other measures, indicating an association between worse erectile functioning and increased anxiety, depression and sexual distress.

Table S4

Correlations between MAIA subscales and PE at each measurement (*N* = 50)

|  | Premature ejaculation | | | |
| --- | --- | --- | --- | --- |
| MAIA subscale | Pre-treatment | Post-treatment | 3 months | 6 months |
| Noticing | -.068 | -.175 | -.279* | -.256 |
| Not-distracting | -.190 | -.230 | -.355* | -.444** |
| Not-worrying | -.004 | .207 | .066 | -.043 |
| Attention regulation | -.210 | -.233 | -.268 | -.140 |
| Emotional awareness | -.187 | -.142 | -.300* | -.315* |
| Self regulation | -.128 | -.224 | -.235 | -.156 |
| Body listening | -.256 | -.274 | -.289* | -.356* |
| Trusting | -.184 | -.169 | -.192 | -.308* |

Note. MAIA = Multidimensional Assessment of Interoceptive Awareness.

* *p* < .05, ** *p* < .01

Intention-to-treat analyses revealed that correlations between PE and body awareness measures increased at later study measurement time point
